# Factors responsible for remote-frequency masking in children and adultsa)

**DOI:** 10.1121/1.4971780

**Published:** 2016-12-19

**Authors:** Lori J. Leibold, Emily Buss

**Affiliations:** Center for Hearing Research, Boys Town National Research Hospital, Omaha, Nebraska 68131, USA; Department of Otolaryngology/Head and Neck Surgery, The University of North Carolina at Chapel Hill, Chapel Hill, North Carolina 27599, USA

## Abstract

Susceptibility to remote-frequency masking in children and adults was evaluated with respect to three stimulus features: (1) masker bandwidth, (2) spectral separation of the signal and masker, and (3) gated versus continuous masker presentation. Listeners were 4- to 6-year-olds, 7- to 10-year-olds, and adults. Detection thresholds for a 500-ms, 2000-Hz signal were estimated in quiet or presented with a band of noise in one of four frequency regions: 425–500 Hz, 4000–4075 Hz, 8000–8075 Hz, or 4000–10 000 Hz. In experiment 1, maskers were gated on in each 500-ms interval of a three-interval, forced-choice adaptive procedure. Masking was observed for all ages in all maskers, but the greatest masking was observed for the 4000–4075 Hz masker. These findings suggest that signal/masker spectral proximity plays an important role in remote-frequency masking, even when peripheral excitation associated with the signal and masker does not overlap. Younger children tended to have more masking than older children or adults, consistent with a reduced ability to segregate simultaneous sounds and/or listen in a frequency-selective manner. In experiment 2, detection thresholds were estimated in the same noises, but maskers were presented continuously. Masking was reduced for all ages relative to gated conditions, suggesting improved segregation and/or frequency-selective listening.

## I. INTRODUCTION

When adults are asked to detect a tone at a predictable frequency, they tend to direct their attention to that spectral region, weighting energy in that region more highly than energy in neighboring frequency regions (e.g., Scharf *et al.*, 1987; Dai *et al.*, 1991; Schlauch and Hafter, 1991). This frequency-selective listening strategy improves adults' performance for detecting sounds at an expected frequency, while decreasing sensitivity to sounds presented at unattended frequencies (e.g., Dai *et al.*, 1991). Infants and young children appear to listen less selectively in the frequency domain than adults during detection tasks (e.g., Bargones and Werner, 1994; Lutfi *et al.*, 2003). For example, Bargones and Werner (1994) presented infants and adults with an “expected” 1000-Hz tone on 75% of trials and tones at one of two “unexpected” frequencies on the remaining 25% of trials. Adults detected the 1000-Hz tone better than they detected tones at the unexpected frequencies, but infants detected tones at expected and unexpected frequencies equally well. These findings are consistent with the idea that adults listen selectively in the frequency domain, but infants listen over a broad range of frequencies.

It has been suggested that infants may listen in an unselective way in order to learn the important cues of speech across a variety of different listening contexts (e.g., Werner, 2007). Findings of studies examining children's speech perception are in general agreement with this hypothesis, and suggest that the use of an unselective listening strategy extends into the school-age years. For example, the perceptual weighting patterns applied to various speech parameters appear to differ between children and adults (e.g., Nittrouer, 1996; Nittrouer *et al.*, 1998). While 4-year-olds are more influenced by global and dynamic speech cues such as formant transitions when they are asked to categorize fricatives, 7-year-olds and adults tend to rely on more detailed cues such as the spectra of the noise (reviewed by Nittrouer, 2006).

One consequence of unselective listening in the frequency domain is that infants and children are often susceptible to auditory masking in the presence of competing sounds that produce little or no masking for adults (e.g., Werner and Bargones, 1991; Allen and Wightman 1995; Leibold and Neff, 2011). For example, infants (Werner and Bargones, 1991) and 4- to 6-year-old children (Leibold and Neff, 2011) exhibit masking in the context of pure-tone detection when a remote-frequency band of noise is present. Werner and Bargones (1991) measured thresholds for detection of a 1000-Hz tone in quiet and presented with a 4000–10 000 Hz broadband noise. Listeners were 6-month-old infants and young adults. In separate masker conditions, the overall level of the masker was either 40 or 50 dB sound pressure level (SPL). Regardless of masker level, the average masking effect for infants was about 10 dB, compared with less than 2 dB for adults.

Leibold and Neff (2011) observed significant, albeit smaller, remote-frequency masking effects in 4- to 6-year-old children. Using a two-interval-forced-choice (2IFC) paradigm, detection thresholds for a 1000-Hz tone presented in quiet or in the presence of a 4000–10 000 Hz band of noise were estimated in 4- to 6-year-old children, 7- to 9-year-old children, and adults. Thresholds were elevated by 3.5 dB in the presence of the remote-frequency noise for 4- to 6-year-olds compared to thresholds in quiet. In contrast, no systematic masking effects were observed for 7- to 9-year-olds or adults. Consistent with the results reported by Werner and Bargones (1991) for infants, no difference in masking was observed between 40- and 60-dB-SPL maskers. These results suggest that the ability to segregate and selectively attend to a pure tone in the presence of remote-frequency noise remains immature into the early school-age years. While there are published data from adults showing remote-frequency masking with a two-octave target/masker separation (reviewed by Patra *et al.*, 2011), those effects are typically restricted to masker levels greater than 80 dB SPL.

In combination with evidence that peripheral frequency resolution is adult-like by at least 3 months following full-term birth (e.g., Abdala and Keefe, 2012), the observation that thresholds for infants and children remain unchanged despite a 10–20 dB increase in masker level (Werner and Bargones, 1991; Leibold and Neff, 2011) supports the hypothesis that remote-frequency masking effects during infancy and early childhood reflect increased susceptibility to centrally based *informational* masking, rather than peripherally based *energetic* masking. While energetic masking can be viewed as the result of overlapping signal/masker excitation on the basilar membrane, informational masking reflects limitations in central auditory processing (e.g., Kidd *et al.*, 1994; Durlach *et al.*, 2003). Informational masking is often described as a failure to separate sounds into distinct auditory objects (e.g., Kidd *et al.*, 1994; Neff, 1995; Durlach *et al.*, 2003); considerable reductions in informational masking have been observed for adults when acoustic cues that facilitate signal/masker segregation are provided (e.g., Kidd *et al.*, 1994; Neff, 1995; Arbogast *et al.*, 2002). Informational masking has also been associated with the degree of perceptual similarity between the signal and masker; informational masking is greatest when the signal and masker are perceptually similar (e.g., Kidd *et al.*, 2002; Durlach *et al.*, 2003). Note that most acoustic cues shown to influence perceptual similarity also play an important role in sound segregation (e.g., Bregman, 1990).

There has been considerable interest over the past decade in determining the extent to which children benefit from the introduction of acoustic cues shown to promote perceptual segregation and reduce informational masking in adults (e.g., Wightman *et al.*, 2003; Hall *et al.*, 2005; Leibold and Neff, 2007). Findings from these studies indicate that children effectively use many, but not all, of the same acoustic cues that benefit adults (e.g., Wightman *et al.*, 2003; Hall *et al.*, 2005; Leibold and Neff, 2007). For example, delaying the onset of a pure-tone signal relative to the onset of a multi-tonal masker is an effective cue for both adults and school-age children (e.g., Hall *et al.*, 2005; Leibold and Neff, 2007). In contrast, presenting a pure-tone signal and multi-tonal masker to opposite ears nearly eliminates masking in adults, but has little or no effect on thresholds in 4- to 5-year-olds (Wightman *et al.*, 2003).

Increased susceptibility to informational masking produced by remote-frequency noise in infants and young children is not restricted to psychoacoustic stimuli. The presence of remote-frequency maskers appears also to interfere with infants' and children's speech perception abilities (e.g., Polka *et al.*, 2008; Newman *et al.*, 2013; Wightman *et al.*, 2010). For example, Newman *et al.* (2013) used a preferential looking time procedure to assessed masked speech recognition in 7- to 9-month-olds. Target speech was composed of multiple repetitions of either the infant's own name or a different name that had the same stress pattern as the child's name. The masker was a one-half octave band of noise that either overlapped in frequency with the target speech (center frequency = 1000 Hz) or was spectrally distinct from the target speech (center frequency = 8000 Hz). Each completed testing in four conditions: (1) infant's own name in on-frequency noise, (2) infant's own name in off-frequency noise, (3) different name in on-frequency noise, and (4) different name in off-frequency noise. The signal-to-noise ratio (SNR) used for all four conditions was +10 dB. Infants listened longer to their own name than a different name in the off-frequency noise masker, but this preference was not observed in the on-frequency noise masker. These data provide evidence of at least a rudimentary ability to segregate and selectively attend to target speech in the presence of off-frequency noise.

Although considerable published data indicate that infants and children have more difficulty than adults in segregating and/or selectively attending to relevant signals in the presence of competing sounds, the specific features of the competing sounds or characteristics of the listener that account for this increased difficulty are not well understood. The two experiments reported here examined the influence of three stimulus factors that could impact child-adult differences in susceptibility to remote-frequency masking: (1) masker bandwidth, (2) spectral separation of the signal and masker, and (3) gated versus continuous masker presentation. The effect of masker bandwidth was evaluated by comparing the masking produced by a wideband (4000–10 000 Hz) and a narrowband (4000–4075 Hz) noise. These bands span 8.2 and 0.2 equivalent rectangular bandwidths (ERBs) (Glasberg and Moore, 1990), respectively. For children younger than 7 years of age, it was predicted that less masking would be produced by the wideband compared to the three narrowband maskers. This prediction was based on the hypothesis that informational masking is influenced by the perceptual similarity between the target and masker (e.g., Durlach *et al.*, 2003); the narrowband noise masker was expected to sound more like the pure-tone signal than the broadband noise masker in the dimension of pitch. The effect of spectral proximity between the signal and masker was examined by comparing the masking produced by a narrowband masker one octave (4000–4075 Hz) and a narrowband masker two octaves (8000–8075 Hz) above the signal frequency. The relative spectral relationship between the signal and masker was evaluated by comparing the masking produced by a narrowband masker two octaves higher in frequency (8000–8075 Hz) and a narrowband masker two octaves lower in frequency (425–500 Hz) than the 2000-Hz signal. Masking effects were expected to be similar across the three narrowband maskers. This prediction was based on the hypothesis that children younger than 7 years of age listen unselectively in the frequency domain during detection (e.g., Leibold and Neff, 2011; Lutfi *et al.*, 2003), and on observations that children older than 7 years of age and adults show little or no masking in the presence of remote-frequency bands of noise (Leibold and Neff, 2011). The effect of gated versus continuous masker presentation was tested by comparing children's and adults' susceptibility to remote frequency masking with a gated (experiment 1) or a continuous (experiment 2) masker presentation. The expectation was that masking, when present, would be reduced for all listeners in the continuous relative to the simultaneously gated masker conditions, consistent with improved signal/masker segregation and frequency-selective listening.

## II. EXPERIMENT 1: GATED MASKER PRESENTATION

The influence of masker bandwidth and spectral separation of the signal and masker on child-adult differences in susceptibility to remote-frequency masking was examined by estimating thresholds for a 500-ms 2000-Hz pure tone in quiet and in each of four, remote-frequency noise conditions. In the first experiment, the masker was 500 ms in duration, such that it gated on and off simultaneously with the pure-tone signal (when present). Based on the results of previous studies (Werner and Bargones, 1991; Leibold and Neff, 2011), the expectation was that children younger than 7 years of age would be susceptible to masking in all four masker conditions, but that no masking would be observed for children older than 7 years of age or adults.

### A. Methods

#### 1. Listeners

Twenty-one children (4.2–10.5 years) and 10 adults (18.4–26.3 years) participated in experiment 1. The children were divided into two groups based on age. Eleven children were younger than 7 years of age (4.2–6.9 years, mean = 5.9 years) and 10 children were older than 7 years of age (7.2–10.5 years, mean = 8.6 years). The rationale for including these two age groups of children is that Leibold and Neff (2011) observed that school-age children younger than 7 years of age are susceptible to remote-frequency masking by a 4000–10 000 Hz noise band, but children older than 7 years of age and adults are not. All listeners had normal hearing, defined as pure-tone thresholds of 20 dB hearing level (HL) or less at octave frequencies 250–8000 Hz bilaterally (re: ANSI, 2010). Exclusion criteria included known developmental delays, a history of hearing problems, previous experience listening in psychophysical studies, and reported chronic middle ear disease. One additional child was tested (4.8 years), but this listener's data were excluded because of an excessively high threshold for the 2000-Hz signal presented in quiet (33 dB SPL) in the forced-choice task.

#### 2. Stimuli and apparatus

In all conditions, the signal was a 500-ms, 2000-Hz pure tone. The masker was a 500-ms, 60-dB-SPL band of noise, presented simultaneously with the signal (when present). The signal and maskers were ramped on and off with 20-ms raised-cosine ramps. The noise masker was filtered in one of four frequency bands: (1) 4000–10 000 Hz, (2) 4000–4075 Hz, (3) 8000–8075 Hz, or (4) 425–500 Hz. Masker bands were generated in matlab by transforming Gaussian noise into the frequency domain, setting components outside the pass-band to a magnitude of zero, and transforming the result back into the time domain. A novel 10.7-s masker sample was generated at the outset of each threshold estimation track. This array was loaded into a real-time processor (RP2; TDT) running at 24 414 Hz. This circuit controlled signal generation, as well as gating of the signal and masker. The summed stimulus was routed through a headphone buffer (HB7; TDT) to the right channel of a pair of circumaural headphones (HD25; Sennheiser).

#### 3. Procedure

Listeners sat in front of a video monitor inside a double-walled, sound attenuating booth (IAC). Stimuli were presented in a three-alternative forced choice, with 500-ms inter-stimulus intervals. On each trial, listeners were presented with a visual display consisting of frogs. One frog's mouth opened during each 500-ms presentation interval, and the listener's task was to select the interval associated with the signal. Adults and children older than 7 years of age indicated their response via a computer mouse. Younger children pointed to the selected frog, and an experimenter inside the booth entered the choice using a computer mouse. Visual feedback followed each listener response. This feedback consisted of a brief animation showing the frog associated with the signal interval catching a fly.

Detection thresholds for the 2000-Hz signal were measured adaptively in quiet and in the presence of each of the four filtered noises. The first threshold estimation track in each condition started at 30 dB SPL. Each subsequent track began with a signal level that was approximately 10 dB above the threshold previously obtained in that condition. The first trial in each track was an orientation trial, in which the signal was presented in the second interval. Orientation trials continued until the correct interval was selected (interval 2); the threshold estimation track began on the following trial. Signal thresholds were determined adaptively using a two-down, one-up stepping rule, estimating the signal level associated with 70.7% correct (Levitt, 1971). The initial step size was 4 dB. The step-size was reduced to 2 dB after the second track reversal. A track continued until eight reversals were obtained. The signal levels at the last six reversals were averaged to obtain an estimate of threshold. Two such estimates were obtained from each listener. A third estimate was obtained if the first two differed by more than 6 dB; this occurred in at least one of the four masker conditions for 8/11 children younger than 7 years of age, 7/10 children older than 7 years of age, and 2/10 adults. No listener required three estimates in all conditions. Additional estimates were required in 45% of conditions for younger children, 29% of conditions for older children, and 2% of conditions for adults.

The mean of all estimates collected for each listener in each condition is reported below. Thresholds were obtained in a different random order for each listener. Adults completed the experiment in a single 1-h session. Children typically completed the experiment in two 1-h sessions, with frequent breaks.

### B. Results

Individual and group average thresholds in quiet are provided in Table I. Quiet thresholds tended to be lower for adults and children older than 7 years of age than for children younger than 7 years of age, with means of 3.9, 5.9, and 11.5 dB SPL, respectively. Levene's test for equality of variance was not significant [*F*(2,28) = 3.00; *p* = 0.07]. A one-way analysis of variance (ANOVA) indicated a significant difference in quiet threshold across the three age groups [*F*(2,28) = 9.69; *p < *0.01; *η^2^_partial_* = 0.41]. *Post hoc* testing (Scheffe, using a criterion of *p* < 0.05) indicated quiet thresholds for the younger children were significantly higher than for the older children (*p* = 0.016) or for adults (*p* = 0.001). Thresholds in quiet were not significantly different for older children and adults (*p* = 0.56). Considering data obtained from all 21 children, the correlation between threshold in quiet and the logarithm of age was statistically significant (*r* = −0.54; *p* < 0.01; one-tailed).

**TABLE I.** Threshold in quiet (dB SPL) and amount of masking (dB) in each filtered noise is shown for individual children <7 years, children >7 years, and adults. The age of each listener is given in years:months. The group average estimates and ± one standard error of the mean (se) are also listed.

		Amount of masking (masked-quiet threshold in dB)			
Listener (age in years:months)	Quiet threshold (dB SPL)	4000–10 000 Hz (8.2 ERB)	4000–4075 Hz (0.2 ERB)	8000–8075 Hz (0.1 ERB)	425–500 Hz (1.0 ERB)
Children <7 years					
4:2	22.67	14.23	27.67	18.67	16.22
4:10	11.17	39.06	59.06	64.11	42.17
5:1	12.00	5.00	9.45	15.89	−2.50
5:4	6.83	38.72	14.50	44.39	29.39
5:7	10.33	7.17	5.51	1.17	4.34
6:2	0.50	4.67	10.83	5.00	11.83
6:3	6.50	2.00	3.00	−4.00	0.34
6:6	10.17	0.17	11.28	4.84	1.84
6:8	17.34	1.83	16.11	7.22	5.00
6:11	14.67	0.50	1.22	−3.33	1.33
6:11	13.89	6.78	8.78	−3.22	5.78
Mean	11.46	10.92	15.22	13.70	10.52
(se)	(1.77)	(4.34)	(4.89)	(6.57)	(4.17)
Children >7 years					
7:2	9.00	6.44	4.33	0.34	−1.00
7:10	8.67	−1.00	8.33	3.00	4.78
7:10	0.84	6.00	7.00	3.33	9.17
7:11	8.00	0.84	20.44	−0.78	−3.67
8:4	2.50	1.67	3.50	3.17	4.67
8:7	3.33	3.89	21.34	9.00	3.67
8:11	5.50	0.17	10.06	3.67	12.83
8:11	10.67	1.34	−0.33	−1.67	−3.34
10:4	4.84	4.17	2.67	1.17	3.17
10:6	5.34	8.22	9.78	8.50	3.17
Mean	5.87	3.17	8.71	2.97	3.35
(se)	(1.00)	(0.96)	(2.30)	(1.12)	(1.64)
Adults					
18:5	3.50	3.84	3.00	2.33	2.84
18:7	0.33	−0.33	3.17	1.01	0.34
19:4	3.00	2.84	−0.50	0.83	0.83
20:6	8.50	0.34	9.00	2.33	−0.17
20:7	5.00	4.00	16.67	9.78	5.67
21:4	3.33	3.34	3.84	4.17	1.01
21:8	3.50	2.67	2.83	0.17	1.00
22:2	2.67	3.84	4.84	3.84	2.50
25:5	3.33	−1.00	1.17	1.50	1.17
26:4	5.33	0.34	18.45	9.17	2.17
Mean	3.85	1.99	6.25	3.51	1.74
(se)	(0.67)	(0.61)	(2.04)	(1.07)	(0.53)

Masking was computed for each listener in each masker condition by subtracting the threshold in quiet from the masked threshold. Individual and group average estimates of the amount of masking for each of the four filtered noise conditions are provided in Table I. Substantial masking of the 2000-Hz signal by the 4000–10 000 Hz masker was evident in the data obtained from children younger than 7 years of age, but less apparent in the data obtained from children older than 7 years of age or adults. For younger children, the average threshold for the 2000-Hz signal was 10.9-dB higher in the presence of the wideband, remote-frequency masker than their average threshold for the same signal in quiet. In contrast, the average threshold difference for older children and adults was 3.2 and 2.0 dB, respectively. One-tailed t-tests were used to evaluate the prediction that masking is larger in the youngest age group; results indicated a non-significant difference between children younger vs older than 7 years of age (*t* = 1.75, *p* = 0.06), and a significant difference between younger children and adults (*t* = 2.04, *p* = 0.03). These results are consistent with maturation in the amount of remote-frequency masking produced the wide (4000–10 000 Hz) masker. This interpretation gets further support from the observation that there was a significant correlation between log of child age and amount of masking (*r* = −0.53, *p* < 0.01; one-tailed).

**FIG. 1.**
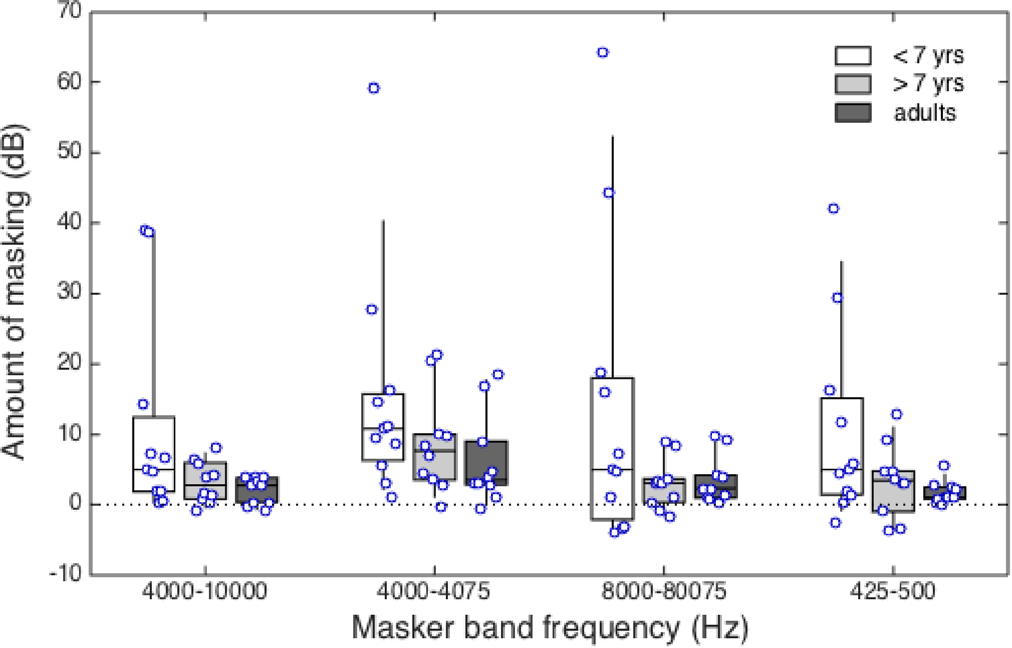
(Color online) Estimates of masking in the presence of the gated, remote-frequency maskers are shown for younger children (<7 years, open boxes), older children (>7 years, light grey boxes), and adults (dark grey boxes). Data are plotted as a function of masker band condition. The horizontal line within each box represents the median value, boxes span the interquartile range (25th–75th percentile), and vertical lines span the 10th to the 90th percentiles. Circles show amount of masking for individual listeners.

Figure 1 summarizes estimates of masking for younger children (<7 years, open boxes), older children (>7 years, light grey boxes), and adults (dark grey boxes), plotted as a function of masker band condition. The horizontal line within each box represents the median value, boxes span the interquartile range (25th–75th percentile), and vertical lines span the 10th to the 90th percentiles. Circles show amount of masking for individual listeners. One question of interest is whether masking was significantly greater than zero across masker type and listener age group. A set of 12 one-sample t-tests was performed to evaluate this question. Of these, ten indicate a significant difference. A non-significant result was obtained for children younger than 7 years of age tested with the 8000–8075 Hz masker (*p* = 0.06) and children older than 7 years of age tested with the 475–500 Hz masker (*p* = 0.07).

While masking tended to be positive for all groups in all maskers, this effect appears to be larger for the 4000–4075 Hz masker than for the other maskers. Considering all four masker conditions, greater masking was observed in the younger children. A repeated-measures ANOVA was performed, with three levels of Group (children <7 years, children >7 years, adults) and four levels of Masker (4000–10 000 Hz, 4000–4075 Hz, 8000–8075 Hz, 425–500 Hz). Mauchly's test of sphericity was significant [*W*(5) = 0.53, *p < *0.01], so Greenhouse-Geisser corrections were applied. There was a significant main effect of Masker [*F*(2.3,63.4) = 5.75, *p < *0.01; *η^2^_partial_* = 0.17], indicating that amount of masking differed across the remote-frequency masker conditions. Neither the main effect of Group [*F*(2,28) = 2.85, *p* = 0.08; *η^2^_partial_* = 0.17] nor the interaction between Masker and Group were significant [*F*(4.5,63.4)= 0.35, *p* = 0.87; *η^2^_partial_* = 0.02].

Given absence of a significant interaction between Masker and Group, continuous effects of child age were evaluated with respect to the mean amount of masking across all four masker conditions. The correlation between log of child age and mean amount of masking was significant (r = −0.55, p < 0.01). This result may appear inconsistent with the failure to find a significant effect of age group in the previous analysis. Note, however, that the relatively small sample size may have reduced the statistical power needed to detect a significant effect of Group. In addition, collapsing results across 4-to-6 years olds could obscure early development. This can be observed in the individual data plotted in Fig. 1, where the open circles within each age group are ordered according to listener age. Within the data for children under 7 years of age, thresholds for the 4-year-olds (left-most points) tended to be higher than those for the 6-year-olds (right-most points).

The main effect of Masker was further evaluated with three planned comparisons (*t* tests, two-tailed, using a criterion of *p* < 0.05): (1) 4000–4075 vs 4000–10 000 Hz, (2) 4000–4075 vs 8000–8075 Hz, and (3) 8000–8075 vs 425–500 Hz. Data were collapsed across the three age groups. The effect of masker bandwidth was assessed by comparing the masking produced by the 4000–10 000 Hz (8.2 ERB) and 4000–4075 Hz (0.2 ERB) maskers. The narrowband masker produced significantly more masking than the wideband masker [*t*(30) = −2.94; *p* < 0.01]. On average, the difference in amount of masking for the 2000-Hz signal in the narrowband compared to the wideband masker was 4.3 dB for children younger than 7 years of age, 5.5 dB for children older than 7 years of age, and 4.3 dB for adults. The effect of spectral proximity between the signal and remote-frequency masker was evaluated by comparing the masking produced by the 4000–4075 Hz (one octave above the signal frequency) and 8000–8075 Hz (two octaves above the signal frequency) maskers. The 4000–4075 Hz masker produced significantly greater masking than the 8000–8075 Hz masker [*t*(30)= 2.23; *p* < 0.05]. On average, the difference in amount of masking for the 2000-Hz signal in the 4000–4075 Hz masker compared to the 8000–8075 Hz masker was 1.5 dB for children younger than 7 years of age, 5.7 dB for children older than 7 years of age, and 2.7 dB for adults. Masking was comparable for narrowband maskers two octaves above (8000–8075 Hz) and two octaves below (425–500 Hz) the signal frequency [*t*(30) = 1.27; *p* = 0.21]. That is, after spectral proximity was taken into account, there was no evidence that high-frequency maskers were more effective than low-frequency maskers.

One striking characteristic of the dataset was the large range of estimates of masking. While this range was greatest within the group of younger children, suggesting age-related changes in susceptibility to remote-frequency masking, substantial individual differences were observed even for adults. For example, amount of masking in the 4000–4075 Hz masker spanned a range of 58 dB for children younger than 7 years of age, 22 dB for children older than 7 years of age, and 19 dB for adults. One remarkable finding was that most individual listeners, including the majority of adults, showed 3 dB or more masking in the presence of the 4000–4075 Hz narrowband noise. Exceptions were one younger child (6.9 years), two older children (8.9 and 10.3 years), and three adults. Masking tended to be largest for the 4000–4075 Hz narrowband masker than for the other masker conditions; this was the case for 6 of 10 adults, 6 of 10 children older than 7 years of age, and 6 of 11 children younger than 7 years of age.

### C. Discussion

Consistent with findings reported by Leibold and Neff (2011), the presence of remote-frequency noise produced substantial masking of a 2000-Hz signal in 4- to 6-year-olds. There is compelling evidence that the cochlea is fully developed and functionally mature by at least 3 months of age following term birth (e.g., Kalluri and Abdala, 2015), the compressive nonlinearity of the basilar membrane is independent of age (e.g., Abdala and Dhar, 2012), and effects of medial efferent stimulation on cochlear tuning are the same for children and adults (e.g., Mishra and Dinger, 2016). Thus, this remote-frequency masking effect observed for children younger than 7 years of age is unlikely to be the consequence of immature peripheral encoding. Alternatively, the more parsimonious explanation is that young school-age children are susceptible to remote-frequency masking because of immature central auditory processing, such as a limited ability to perceptually segregate the pure-tone signal and the remote-frequency masker and/or selectively attend to sound presented at a specific frequency.

It has been suggested that infants' and children's tendencies to integrate (rather than segregate) sounds and their use of unselective listening strategies facilitate speech and language learning (e.g., Werner, 2007; Jones *et al.*, 2015). For example, Jones *et al.* (2015) suggest that developmental effects in auditory selective attention reflect an initial strategy used by children to “exclude as little sensory information as possible.” While this general approach may facilitate learning about speech and other important sounds, the present results are consistent with previous data indicating that such a strategy increases children's vulnerability to the detrimental effects of competing background sounds.

The effect of masker bandwidth was evaluated for all three age groups by comparing the masking produced by a wideband of noise with energy distributed over 8.2. ERBs (4000–10 000 Hz) and a narrowband of noise with energy distributed over 0.2 of an ERB (4000–4075 Hz) noise. Significantly greater masking was observed in the presence of the 4000–4075 Hz compared with the 4000–10 000 Hz masker. While the narrowband masker had greater energy closer to the signal frequency than the wideband masker, recall the 2000-Hz signal/masker separation. Thus, it is highly unlikely that spread of excitation could be introducing on-frequency masking in either case. Moreover, previous data indicate that remote-frequency masking of a 1000-Hz signal is not sensitive to the level of a 4000–10 000 Hz noise masker (e.g., Werner and Bargones, 1991; Leibold and Neff, 2011). Although the present study evaluated detection of a 2000-Hz signal, the relatively high spectrum level in the region of 4000 Hz for the narrowband masker is unlikely responsible for this masker bandwidth effect. One explanation for this result is that the narrowband masker is more likely to produce informational masking than a wideband masker due to increased target-masker similarity (e.g., Durlach *et al.*, 2003). That is, the narrowband masker may have been more easily confused with the pure-tone signal than the broadband noise because it had a pitch-like quality. However, an alternative argument can be made that the greater envelope fluctuations inherent in the narrow-band relative to the broadband noise masker may have provided listeners with a salient segregation cue.

The effect of spectral proximity between the signal and masker was evaluated by comparing the masking produced by a 4000–4075 Hz and an 8000–8075 Hz narrowband of noise. Significantly greater masking was observed for the 4000–4075 Hz masker than for the 8000–8075 Hz masker. No significant masking was observed between the 425–500 and 8000–8075 Hz maskers, indicating that relatively low and relatively high-frequency narrowband maskers are equally effective when the frequency separation between the signal and remote-frequency masker is equated. This pattern of results suggests that the spectral proximity of the signal and masker plays an important role in masking for listeners of all ages, even when the signal and masker excite different populations of neurons in the peripheral auditory system.

One *a priori* prediction was that masking effects would be similar across the three narrowband masker conditions for the youngest listeners, based on the hypothesis that children younger than 7 years of age listen unselectively in the frequency domain (e.g., Leibold and Neff, 2011; Lutfi *et al.*, 2003). The expectation was that, if children younger than 7 years of age use a truly unselective listening strategy, the frequency proximity between the target and masker would not influence masking for this age group. The present results do not support this prediction. We are unaware of other studies in the literature that have evaluated remote-frequency masking in children as a function of signal/masker frequency proximity. However, Greenberg *et al.* (1970) evaluated frequency-selective listening in 6- to 8-year-old children and adults using the probe-signal method of Greenberg and Larkin (1968). The task was to detect fixed-frequency pure tones (850, 925, 1000, 1075, and 1150 Hz) presented in continuous broadband noise. The levels of each pure tone were selected to achieve equal detection performance when presented alone in a block of trials. To assess frequency-selective detection, listeners were tested in conditions in which the signal was 1000 Hz on the majority of trials (∼70%) and one of the other frequencies (850, 925, 1075, or 1150 Hz) on the remaining trials (∼30%). Both children and adults showed greater sensitivity to tones at the expected frequency and reduced sensitivity with increasing deviation from the expected frequency. In addition, an examination of the individual data reported by Greenberg *et al.* (1970) show flatter functions for most children than adults.

An unexpected finding of the present study was that listeners of all ages, including adults, were susceptible to remote-frequency masking by a 4000–4075 Hz noise band. The target signal was a 2000-Hz tone; it is unlikely that the remote-frequency noise band and the pure-tone signal produced overlapping peripheral excitation on the basilar membrane sufficient to elevate detection thresholds (e.g., Chen *et al.*, 2011).^1^ Moreover, while the maskers differed in loudness as estimated using the excitation model proposed by Chen *et al.* (2011), the estimated loudness for the 4000–4075 Hz masker (2.62 sones) was less than for the 4000–10 000 Hz masker (2.69 sones).^2^ This observation supports the idea that the effectiveness of a remote-frequency masker is not due entirely to its loudness.

In contrast to many previous psychoacoustic studies involving adults, one criterion for inclusion in this experiment was that listeners have no previous experience in psychoacoustic experiments. In addition, thresholds for each condition were estimated based on 2–3 runs per condition to ensure consistency with the procedures used to test children. In order to investigate the possibility that the masking for inexperienced adults would not be observed for experienced listeners with training, supplemental data were collected on an additional group of 10 adults (20.8–43.0 years). Each new adult listener had previously completed a minimum of 10 h of testing in similar psychoacoustic experiments. The stimuli and procedures were identical to those used to test children and untrained adults, except that six estimates of threshold were obtained from each listener for each condition across three, 1-h sessions. The first two estimates were considered practice and the mean of the last four estimates of threshold for each condition was computed.

The adults with extensive listening experience showed less masking than inexperienced adults. Nonetheless, a similar trend in performance across the four masker conditions was observed for the experienced and inexperienced listeners. Specifically, greater masking was observed for both groups of adults with the 4000–4075 Hz masker compared with the other three maskers. The average amount of masking for experienced adults was 0.6 dB in the 4000–10 000 Hz masker (range = −0.8 to 3.1 dB), 1.7 dB in the 4000–4075 Hz masker (range = −0.2 to 4.2 dB), 0.8 dB in the 8000–8075 Hz masker (range = −1.8 to 2.6 dB), and 0.1 dB in the 425–500 Hz masker (range = −1.5 to 1.5 dB). A repeated-measures ANOVA was performed, with the between-subjects factor of Group (experienced adults, inexperienced adults) and the within-subjects factor of Masker (4000–10 000 Hz, 4000–4075 Hz, 8000–8075 Hz, 425–500 Hz). Mauchly's test of sphericity was significant [*W*(5) = 0.03, *p* < 0.001], so Greenhouse-Geisser corrections were applied. The analysis revealed a significant main effect of both Masker [*F*(1.2,21.1)= 7.46, *p* < 0.05; *η^2^_partial_* = 0.29] and Group [*F*(1,18) = 7.14, *p* < 0.05; *η^2^_partial_* = 0.28]. The Masker × Group interaction was not significant [*F*(1.2,21.1) = 2.09, *p* = 0.16; *η^2^_partial_*= 0.10]. These results suggest that limited training is not fully responsible for the increased masking observed for listeners in the presence of the narrowband masker most proximal in frequency to the 2000-Hz signal.

## III. EXPERIMENT 2: CONTINUOUS MASKER PRESENTATION

The second experiment tested the hypothesis that gating the signal on and off together produced informational masking effects for many listeners in experiment 1. Previous investigations have demonstrated that both adults (e.g., Neff, 1995) and children (e.g., Hall III *et al.*, 2005) show a marked reduction in informational masking when there is a temporal asynchrony between the signal and masker compared to when the signal and masker are simultaneous gated. The temporal asynchrony is through to aid in sound segregation by increasing the saliency of the signal. Evidence supporting this idea comes from studies showing substantial improvements in pure-tone detection thresholds when the onset of a pure-tone signal is delayed relative to a remote-frequency, multi-tonal masker, but not for relatively long-duration signals when the masker is on-frequency, broadband noise (e.g., Neff, 1995; Leibold and Neff, 2007).

In addition to not providing a segregation cue, simultaneously gating the signal and masker in experiment 1 may have undermined the ability to listen selectively in frequency. Wright and Dai (1994) measured attention filters for adults in a background noise using the probe signal method. In one set of conditions the signal was a 295-ms tone. The masker was a band of noise (0–8000 Hz), with a 20-dB spectrum level, that was either 295 ms in duration or played continuously. When the masker played continuously, attention filters were sharply tuned to the expected signal frequency for all four listeners, such that the attention band resembled an auditory filter. When the masker was the same duration as the signal, attention bands were sharply tuned to the expected frequency for two of the listeners and very broadly tuned for the other two listeners. Greater variability in results with the gated than the continuous masker prompted Wright and Dai (1994) to suggest that listeners employed a more unstable listening strategy with the gated masker. If frequency-selective listening in adults is more difficult in a gated than a continuous masker, then one might predict that young children would have even more difficulty with a gated masker.

### A. Methods

#### 1. Listeners

Nine 4- to 6-year-olds (4.4–6.8 years), 10 7- to 8-year-olds (7.0–8.7 years), and 10 adults (19.1–29.5 years) participated in experiment 2. None of the listeners in experiment 2 were tested in experiment 1. All listeners had normal hearing (re: ANSI, 2010). Exclusion criteria included known developmental delays, a history of hearing problems, previous experience listening in psychophysical studies, and reported chronic middle ear disease. Data from one additional child (4.3 years) were excluded because this listener was unable to reliably perform the forced-choice task in quiet.

#### 2. Stimuli, apparatus and procedure

The stimuli, apparatus and procedure were as in experiment 1, with the exception that maskers were presented continuously throughout a block of trials. Filtered noises were generated as in experiment 1; this procedure resulted in a 10.7-s sample of noise that could be repeated without discontinuity at the beginning and the end of the array. Two threshold estimates were obtained from each listener in each of the four masker conditions, and a third was obtained when the first two differed by more than 6 dB. Additional estimates were required in 14% of conditions for 4- to 6-year-olds, 43% of conditions for 7- to 8-year-olds, and 10% of conditions for adults. The mean of all estimates collected for each listener in each condition is reported below. Thresholds were obtained in a different random order for each listener. Adults completed the experiment in a single 1-h session. Children typically completed the experiment in two 1-h sessions, with frequent breaks.

### B. Results

Table II shows individual and group average thresholds in quiet. In contrast to experiment 1, a similar range of quiet thresholds was observed between the three age groups of listeners. Quiet thresholds ranged from −2.8 to 14.7 dB SPL for adults (mean = 6.3 dB SPL), from 1.2 to 13.3 dB SPL for children older than 7 years of age (mean = 6.9 dB SPL), and from 2.7 to 14.4 dB SPL for children younger than 7 years of age (mean = 8.9 dB SPL). Levene's test of equality of error variances was not significant [*F*(2,26) = 2.33; *p = *0.12]. Results of a one-way ANOVA indicated no significant difference in quiet threshold between the three age groups [*F*(2,26) = 0.91; *p = *0.42; *η^2^_partial_* = 0.07]. Also unlike the results for experiment 1, no significant correlation was observed between quiet threshold and the logarithm of child age (*r* = −0.32; *p* = 0.09; one-tailed).

**TABLE II.** Threshold in quiet (dB SPL) and amount of masking (dB) in each filtered noise is shown for individual children <7 years, children >7 years, and adults. The age of each listener is given in years. The masker was played continuously. The group average estimates and ± one standard deviation (SD) are also listed.

		Amount of masking (masked-quiet threshold in dB)			
Listener (age in years)	Quiet threshold (dB SPL)	4000–10 000 Hz (8.2 ERB)	4000–4075 Hz (0.2 ERB)	8000–8075 Hz (0.1 ERB)	425–500 Hz (1.0 ERB)
Children <7 years					
4.4	14.39	16.83	15.66	10.83	8.11
5.3	8.00	2.83	5.33	2.83	3.50
5.5	9.50	−1.00	−2.50	−1.67	−3.17
5.9	2.67	2.17	−0.50	0.67	0.83
6.0	8.33	1.83	0.00	2.33	1.33
6.4	10.50	0.84	0.50	1.17	1.34
6.6	9.50	−0.33	0.34	3.17	0.84
6.8	10.00	1.61	−2.94	−3.17	4.33
6.8	6.83	0.83	1.50	−0.50	0.00
Mean	8.86	2.85	1.93	1.74	1.90
(SD)	(3.14)	(5.38)	(5.68)	(4.01)	(3.15)
Children >7 years					
7.0	7.42	0.50	0.50	−0.33	1.01
7.1	1.50	1.00	2.83	2.00	0.17
7.2	7.50	3.61	0.44	1.83	1.56
7.8	10.00	−0.33	0.66	−0.95	1.84
7.8	7.39	−2.89	−4.72	−2.56	−5.39
7.9	11.33	2.39	5.39	0.55	3.17
8.4	4.50	−1.67	0.17	0.67	0.00
8.5	5.17	13.67	2.39	0.17	0.34
8.7	1.17	1.83	0.17	0.83	−3.50
8.7	13.33	1.17	1.67	1.17	3.17
Mean	6.93	1.93	0.95	0.34	0.24
(se)	(3.97)	(4.54)	(2.58)	(1.36)	(2.75)
Adults					
19.1	2.67	1.50	2.50	3.17	3.17
19.2	−2.84	3.34	2.34	3.17	0.17
19.7	8.17	2.00	1.17	2.33	2.83
19.8	14.67	1.00	0.34	−1.33	−0.33
21.9	12.17	−3.33	−1.34	0.84	−1.67
22.5	3.17	0.50	0.67	−0.33	−2.11
24.2	9.50	−2.67	0.17	−2.50	−1.84
24.8	9.17	−1.17	2.17	0.28	0.17
25.6	2.50	−0.34	−0.17	1.00	1.83
29.5	3.84	1.50	0.50	2.00	2.17
Mean	6.30	0.23	0.84	0.86	0.44
(se)	(5.31)	(2.11)	(1.22)	(1.89)	(1.97)

**FIG. 2.**
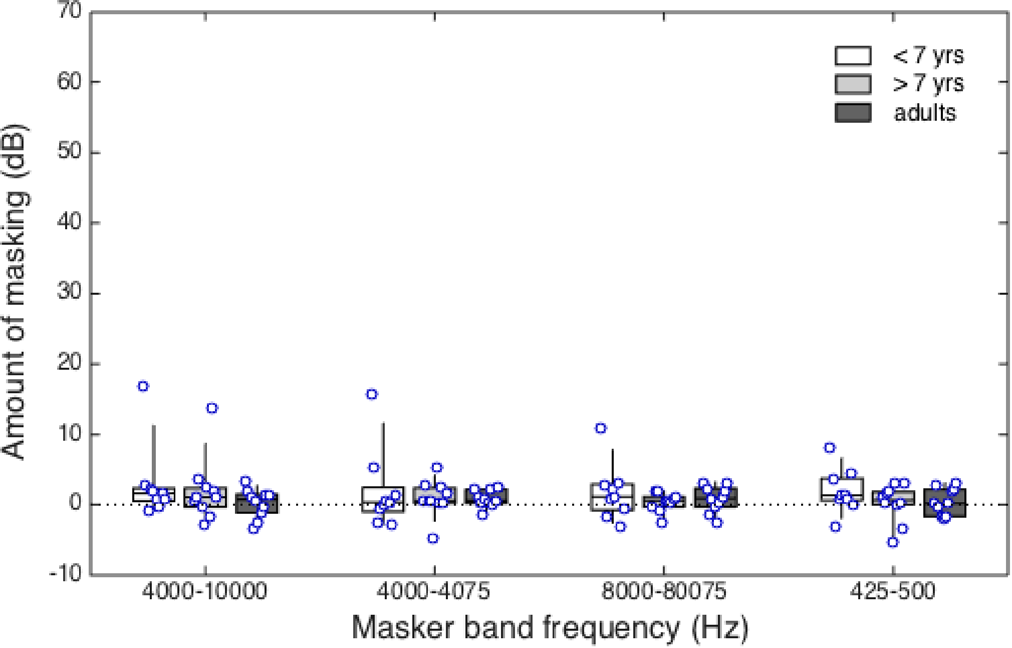
(Color online) Estimates of masking in the presence of the continuous, remote-frequency maskers are shown for younger children (<7 years, open boxes), older children (>7 years, light grey boxes), and adults (dark grey boxes). Data are plotted as a function of masker band condition. The horizontal line within each box represents the median value, boxes span the interquartile range (25th–75th percentile), and vertical lines span the 10th to the 90th percentiles. Circles show amount of masking for individual listeners.

Estimates of masking are shown for the three age groups in Table II. These data are summarized in Fig. 2, following the same format used in Fig. 1. In contrast to the results for experiment 1, little or no masking was observed for either age group of children or for adults when the maskers were played continuously throughout testing.

A repeated-measures ANOVA was performed on amount of masking, with three levels of Group (children < 7 years, children > 7 years, adults) and four levels of Masker (4000–10 000 Hz, 4000–4075 Hz, 8000–8075 Hz, 425–500 Hz). Mauchly's test of sphericity was not significant [*W*(5) = 0.68, *p = *0.09]. The main effect of Masker was not significant [*F*(3,78) = 0.95, *p = *0.42; η^2^_*partial*_ = 0.04], indicating the four filtered noises produced equivalent masking when played continuously throughout testing. The main effect of Group was not significant [*F*(2,26) = 0.75, *p = *0.48; *η*^2^_*partial*_ = 0.06], indicating similar masking across the three age groups of listeners. The interaction between Masker and Group was not significant [*F*(2,26) = 0.03, *p* = 0.97; *η*^2^_*partial*_ = 0.003].

One question of interest is whether masking was significantly different from zero in the continuous masker conditions. Given the absence of significant Masker effects, values were averaged across conditions for each listener. These composite values had a mean of 1.2 dB, which was significantly different from zero [*t*(28) = 2.21, *p* = 0.04, two-tailed]. Within child listeners, there was a correlation between the log of child age and amount of masking (*r* = −0.48, *p* = 0.04). These results indicate that whereas amount of masking was greatly reduced by playing the masker continuously, there is still some indication of an age effect.

Estimates of amount of masking varied extensively across listeners within and across age groups, but the range of individual differences was markedly smaller with the continuous masker presentation than observed in experiment 1 with the gated masker presentation. For example, while amount of masking spanned a range of 58 dB for younger children in the 4000–4075 Hz gated masker (experiment 1), this range was less than 19 dB in the same masker when it was played continuously (experiment 2). Interestingly, the single 4-year-old tested in this experiment showed considerably more masking than all other older listeners, with amount of masking ranging from 8.1 to 16.8 dB.

### C. Discussion

The main result of this experiment is that the effect of signal/masker frequency proximity observed in experiment 1 using a gated masker presentation was not observed when the same maskers were presented continuously, and overall there was substantially less masking in any condition. This finding is consistent with the hypothesis that the 4000–4075 Hz gated noise evaluated in experiment 1 produced informational, rather than energetic masking. This hypothesis is based on findings that listeners tend to group sounds that start and stop at the same time (e.g., Bregman, 1990). Thus, the continuous masker presentation facilitates segregation of the 2000-Hz signal from the remote-frequency noise.

Six-month-old infants tested by Werner and Bargones (1991) experienced an average of 5 dB less masking of a 1000-Hz signal when a 4000–10 000 Hz masker was played continuously through testing than when it was gated. Nonetheless, significant masking was observed for infants with the continuous masker presentation. It is interesting to note in the present dataset that the youngest listener (4.2 years) showed substantially more masking in the continuous remote-frequency noise bands than all other listeners. In combination with the infant data (Werner and Bargones, 1991), this observation raises the question of when in development the ability to segregate and selectively attend to a target signal and disregard a continuously presented remote-frequency masker develops. Future studies involving toddlers and preschoolers are required in order to fully delineate the time course of development for susceptibility to remote-frequency noise masking.

The specific mechanisms responsible for age effects in informational masking produced by gated, remote-frequency bands of noise are not fully understood. Previous interpretations for infants' and children's increased susceptibility to remote-frequency noise have largely focused on contributions of immature selective auditory attention (e.g., Werner and Bargones, 1991; Leibold and Neff, 2011). While the relatively limited number of masker conditions included in the present study prevent a detailed account of the effect of signal/masker frequency separation on detection performance, it is interesting to note that the same general pattern of results was observed across the four masker conditions in experiment 1 for all three age groups. One possible explanation for younger children's increased susceptibility to informational masking in the presence of gated, remote-frequency noise is that they lack the listening experience required to fully segregate and selectively attend to sounds in the absence of the robust onset/offset cue provided by the continuous noise in experiment 2.

There is a literature in adults showing poorer masked tone detection in the presence of a remote-frequency masker when target and masker stimuli are presented simultaneously compared to when listeners are provided with a precursor of the masker alone prior to each trial (e.g., Carlyon, 1989) or when signals are presented with some delay after masker onset (e.g., Wright, 1997). Whereas functional frequency selectivity for brief tones appears to sharpen over time, it is unclear how simultaneous gating affects performance for longer signals, like the 500-ms duration using in the present experiment. Central factors have been implicated in previous demonstrations of these enhancement effects with short signals. These include adaptation, inhibition, suppression, and signal/masker confusion caused by perceptual similarity (e.g., Carlyon, 1989; Nelson and Young, 2010). The relative contributions of effects associated with prior stimulation likely depend on the stimuli and listening conditions (Feng and Oxenham, 2015). While the peripheral auditory system is functionally mature in school-age children, more central processes may not be. There are numerous datasets consistent with maturation of higher order processes, such as auditory stream segregation (e.g., Oh *et al.*, 2001). There are also data on suppression of distortion-product otoacoustic emissions, an indicator of efferent function, indicating that the medial olivo-cochlear reflex is strongest in neonates and declines with each decade of life, from childhood into old age (Konomi *et al.*, 2014). A stronger MOC reflex would be expected to improve performance when a signal is delayed relative to masker onset, but it is unclear how it would contribute to poorer thresholds in the gated condition, as observed in the present data. Considerations like these prompt us to conclude that the most likely factor responsible for the age effect observed here is central processing, related to the ability to segregate concurrent sounds and/or selectively attend in frequency.

## IV. SUMMARY AND CONCLUSIONS

(1) On average, detection threshold for a 2000-Hz signal was elevated by over 10 dB for children younger than 7 years of age in a gated, 4000–10 000 Hz noise band relative to quiet. This result is consistent with previous results observed for infants (Werner and Bargones, 1991) and 4- to 6-year-olds (Leibold and Neff, 2011), indicating a reduced ability of young children to listen in a frequency-selective manner. However, there were marked individual differences in susceptibility to remote-frequency masking in the youngest children, with approximately 1/3 performing like older children and adults.
(2) In gated masker conditions, significantly greater masking was observed for the 4000–4075 Hz masker than for the 4000–10 000 Hz masker. The amount of masking was similar for the 425–500 and 8000–8075 Hz maskers. This pattern of results, observed for all age groups, suggests that the spectral proximity of the signal and masker plays an important role in gated masking, even when the signal and masker excitation do not overlap in the periphery.
(3) One unexpected finding was that older children and adults were susceptible to remote-frequency masking by a gated, 4000–4075 Hz noise band. Supplemental data obtained from ten adults with extensive psychoacoustic listening experience were in agreement with the results observed for the untrained listeners, providing evidence that this masking effect is at least partly resistant to training.
(4) Playing the masker continuously tended to reduce remote-frequency masking compared to results with the gated masker. No effect of signal/masker frequency proximity was observed for any age group with continuous masker presentation. This finding is consistent with the idea that informational masking is responsible for the age and spectral separation effects observed using gated maskers, potentially due to challenges perceptually segregating simultaneously presented sounds.

## Footnotes

^1^ Excitation patterns were calculated for the 2000-Hz pure tone presented with each of the four remote-frequency masker conditions. As expected, the model output showed that the 425–500 Hz narrowband noise resulted in the most peripheral excitation in the frequency region of 2000 Hz. Note, however, that the model output confirmed the amount of peripheral excitation produced by the remote-frequency maskers was substantially lower than for the 2000-Hz signal.

^2^ Estimates of loudness generated from the Chen *et al.* (2001) excitation model were: (1) 2.69 sones for the 4000–10 000 Hz masker, (2) 2.62 sones for the 4000–4075 Hz masker, (3) 0.95 sones for the 425–500 Hz masker, and (4) 1.76 sones for the 8000–8075 Hz masker.
